# Spatial Healthcare Accessibility: A District-Level Analysis of Travel for Outpatient Diabetology in Czechia

**DOI:** 10.3390/healthcare10020395

**Published:** 2022-02-19

**Authors:** Luděk Šídlo, Kateřina Maláková

**Affiliations:** Department of Demography and Geodemography, Faculty of Science, Charles University, 116 36 Prague, Czech Republic; katerina.malakova@natur.cuni.cz

**Keywords:** health services, access to care, public health, outpatient diabetology, rural health care

## Abstract

Assessments of regional differences in the accessibility and capacity of health services often rely on indicators based on data from the permanent residents of a given region. However, a patient does not always use health services in their place of residence. The objective of this article is to evaluate the influence of spatial healthcare accessibility on regional differences in the provision and take-up of health services, using outpatient diabetology in Czechia as a case study. The analysis is grounded in monitoring the differences in the patient’s place of residence and the location of the healthcare provided. Anonymized individual data of the largest Czech health insurance company for 2019 are used (366,537 patients, 2,481,129 medical procedures). The data are aggregated at the district level (LAU 1). It has been identified that regions where patients travel outside their area of residence to access more than half of their healthcare needs are mostly in local/regional centres. Moreover, these patients increase the number of medical services provided in local/regional centres, often by more than 20%, which has been reflected in greater healthcare capacity in these centres. To assess regional differences, it is important to take the spatial healthcare accessibility into account and also consider why patients travel for healthcare. Reasons could be the insufficient local capacity, varied quality of health services or individual factors. In such cases, healthcare actors (health insurance companies, local government etc.) should respond to the situation and take appropriate action to reduce these dissimilarities.

## 1. Introduction

At some point, we all become patients and have to seek out the healthcare services we require. Except in medical emergencies requiring acute care, such as those provided in hospitals, in the vast majority of cases patients visit the medical practice with which they are registered, or select a specific doctor. One can assume that the initial choice of personal doctor is determined by a whole range of factors. Many scholars [1,2,3,4,5] have investigated the determining factors behind the choice of healthcare provider. Their findings indicate that there is no such thing as a ‘typical patient’ when it comes to choosing a doctor; different patients in different situations decide in different ways. Nonetheless, patients tend to favour medical services near their place of residence, rather than choosing from a wider range spread out over a greater distance [2,6].

According to Victoor et al. [1], when patients choose a doctor, they often make their decision based on partial information, or according to their current situation, rather than entering into a complex rational decision making process. Levesque et al. [7] point out that being fully informed as to potential treatments is an important part of ensuring ‘patient centred health care’, while Hoffstedt et al. [8] think it is key to fostering competition among providers, which can lead to better health services. Victoor et al. [1] also note that although people may consider easily accessible information sources important because they enable provider comparisons, in practice not many people use the sources that do exist and even fewer make their decisions based upon them. Instead, previous experience is a key factor in choosing a provider.

Inche Zainal Abidin et al. [4], who focused on patients being treated for diabetes, have described the main factors affecting choice of doctor: age, marital status, education, employment status, income, size of household, comorbidity, family history of diabetes, general family background and perceived severity of the disease. These factors not only affect healthcare-seeking behaviour but also healthcare-utilization behaviour, and the authors emphasize the role played in this by family background and family experience of the disease. Other important factors include interpersonal ones such as atmosphere, physician´s communication style, access to information, continuity of care (stable healthcare teams) and waiting times. According to Victoor et al. [1] these determinants are even more important when the individual making the choice has a low level of education.

Therefore, it is unlikely that patients will always use the healthcare services in the town or region they live in. Those travelling outside their territorial unit for healthcare are either forced to do so for objective reasons (e.g., the lack or complete absence of medical care) or subjective reasons (relying on healthcare services near their place of work, or study, the doctor’s reputation, satisfaction with the provided health care [9,10,11,12,13]. The decision may also depend on the type of healthcare sought [14,15]. When selecting primary healthcare, people often choose a doctor that is geographically close and thereby a short journey away [3]. But when deciding on outpatient specialist care, where patients may only attend examinations a couple of times a year, one can assume that patients take a wider range of factors into consideration, see for example Victoor et al. [1].

The present article focuses on one of the most common types of outpatient specialist care—outpatient diabetology—and compares the patient’s place of residence and the location of healthcare provided. The patient group of interest consists of patients insured by the largest health insurance company in Czechia, a country with a highly developed healthcare system and generally accessible health services. Diabetology services were selected because the incidence of diabetes mellitus is rising in Czechia [16] as well as around the world [17], and will continue to rise, partly because of population ageing, but other factors also play a role [18,19]. Treating diabetes places great pressure on health systems; it is associated with comorbidities and complications that can seriously affect quality of life. Type 2 diabetes mellitus is an avoidable disease and hence the focus is on limiting its prevalence. One such method is prevention, or at least early medical intervention, both of which are linked to healthcare usage rates. The factors affecting diabetes treatment take-up rates are analysed in [20,21,22]. However, the focus of the present analysis is primarily on the spatial accessibility of diabetology healthcare and using the data to accurately assess regional differences in healthcare provision and/or take-up.

## 2. Materials and Methods

A number of studies deal with spatial healthcare accessibility and rely on a geographic information system (GIS) [23,24,25]. Most of them use detailed geographic data on patients and health service providers. The present article is not different in this respect: it uses data from the database belonging to the General Health Insurance Company (GHIC) in Czechia [26]. At the time of writing, the database was the only comprehensive data source of the required depth and detail accessible in Czechia. GHIC is the most popular health insurance company, covering almost 60% of insurance holders. Although GHIC insurance holders tend to be slightly older than other insurance holders, the database is sufficiently robust for an analysis of this type.

Specifically, the data used in this analysis are anonymized individual data on outpatient diabetology care provided to GHIC insurance holders in 2019, sorted by procedure code, health diagnosis and care provider. To the data on each patient, we added data on the patient’s municipal residence (LAU 2), sex and age (as of 31 December 2019). Insurance holders for whom this data was not available were excluded from the dataset. For 2019, there were 1,457 patients who could not be identified with an outpatient diabetology service, representing 0.5% of the total number of unique insurance holders treated, and 0.4% of the total number of medical procedures. As this is an insignificant figure, the overall results are not affected. The final adjusted dataset contains data on 366,537 patients and 2,481,129 medical procedures.

Lastly, healthcare provider data was also used; specifically, sorted anonymized data on the capacity (total and by age categories: under 40, 40–59, 60 and over) of contracted outpatient diabetology providers as of 31 December 2019 and the geographical location of the service. The GHIC data on healthcare providers covers the vast majority of healthcare providers in Czechia because it is legally responsible for healthcare quality and access. For the purposes of this study, we can consider the network of outpatient diabetology providers contracted by GHIC to represent almost the complete picture. The data relates to 549 outpatient diabetology services with a total workload capacity of 423.6 full-time equivalent (FTE) doctors.

Spatial healthcare accessibility can be analysed from various angles. The approach adopted in this paper is based on identifying discrepancies between the patient’s district of permanent residency and the district in which the patient’s healthcare provider is located. The level of detail in the input data also allows for a municipal level analysis (LAU 2). However, there are 6,258 municipalities in Czechia and only 234 of these have outpatient diabetology services, and we would have to focus on these for the spatial accessibility analysis to make sense. We therefore decided to use data aggregated at the next level up; in Czechia that is the district level (LAU 1). There are 77 districts in Czechia and each district has an outpatient diabetology service. Districts have a long history in Czechia and are frequently used as the administrative unit level at which the state administration, local government, and other institutions operate. Each district has a district town that is the local center of the area, providing work, education, and services, including healthcare. GHIC, among others, has its territorial client offices in individual district towns. GHIC is obliged by law to ensure the access to health services for patients in the region of their residence. At the same time, it aims to provide healthcare services, like specialized outpatient services, in the patient’s district of residence, if the characteristics of the health services correspond to this. It cannot be ruled out that a patient who lives near the district border has better access to a doctor in a neighbouring district. However, this is one of the many factors that will always influence the analysis. Even if we were to work with specially created catchment regions [27,28,29], which would more accurately reflect the commuting patterns in the regions, we would still not completely remove the influence of these factors. This issue could be further compounded by, for example, the difference between the patient’s permanent (i.e., officially registered) residence and the usual (i.e., actual) residence. In this respect, these administrative units are well suited to an analysis of this type. In this study, spatial healthcare accessibility is defined as the difference between the patient’s district of residence and the district where the patient accessed the healthcare. One could perform the analysis at the next administrative level up, the regional level (NUTS 3), of which there are 14 in Czechia. However, the NUTS 3 level is less granular and would not enable us to identify the main aspects of spatial healthcare accessibility.

The suitability of the district level for this analysis can be seen in Table 1, which compares the structure of the patient population and medical procedures at the various administrative unit levels. At the municipal level (LAU 2), the data are for patients resident in a municipality containing outpatient diabetology services (57% of the total number of patients). Comparing the LAU 2 and LAU 1 levels, we can see that the patient population structure reliant on outpatient diabetology services in the area of residency (IN) versus outside the area of residency (OUT) is very similar. Only a very small proportion of patients use services both in and outside their area of residency (IN and OUT). At the regional level (NUTS 3), by contrast, we see that only a small proportion of patients use diabetology services outside the region in which they live, and so this level is too general for analysing accessibility. The data in Table 1 also show that the medical procedure structure is very similar to the patient population structure. Furthermore, the medical procedures are clearly divided into those provided in the place of residence and those which are not. There are always patients who access healthcare services within their district of residence and those who access healthcare services in another district. In the analytical section we will therefore consider accessibility, not in terms of patients, but according to the number of procedures performed. That way we can clearly identify the location of the healthcare service vis-à-vis the patient’s place of residence.

## 3. Results

**Figure 1:**
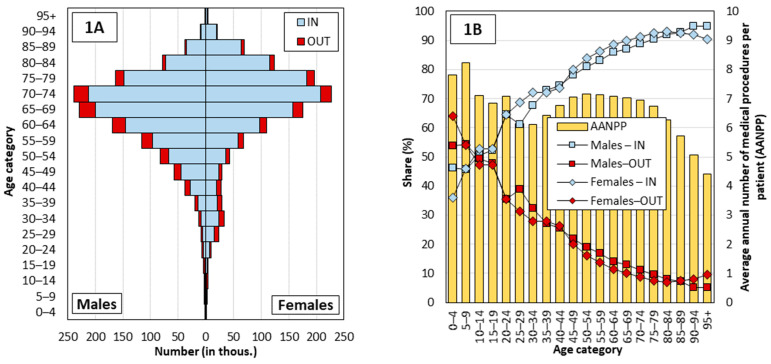
Structure of outpatient diabetology procedures by patient age and gender (**1A**) and by lo-cation of healthcare provision in terms of patient’s district (LAU 1) of residence (**1B**), and average annual number of procedures per patient by age (**1B**); Czechia, 2019, GHIC patients. Note: IN, OUT – see Table 1.

Before turning to the analysis of the regional differences, there is one important aspect affecting spatial healthcare accessibility still to be considered—patient age. In 2019, elderly people constituted most patients attending outpatient diabetology services. Patients aged 60–84 accounted for almost 70% of all procedures performed; by contrast, the under 40s represented only 7% of patients (see Figure 1A). The annual number of procedures per patient did not differ greatly by patient age, except for the oldest age category, and was 6–7 procedures for that year (Figure 1B, bar chart). What does change with age, though, is the proportion of procedures patients access in their district of residence, which rises with age, from around 50% for the lowest age categories to over 90% for the oldest age category, for both genders (Figure 1B). These age differences in healthcare access are important given the long-term variation in the age composition of patients/inhabitants in the districts [30], which largely reflects the district’s appeal, especially among young people. 

**Figure 2:**
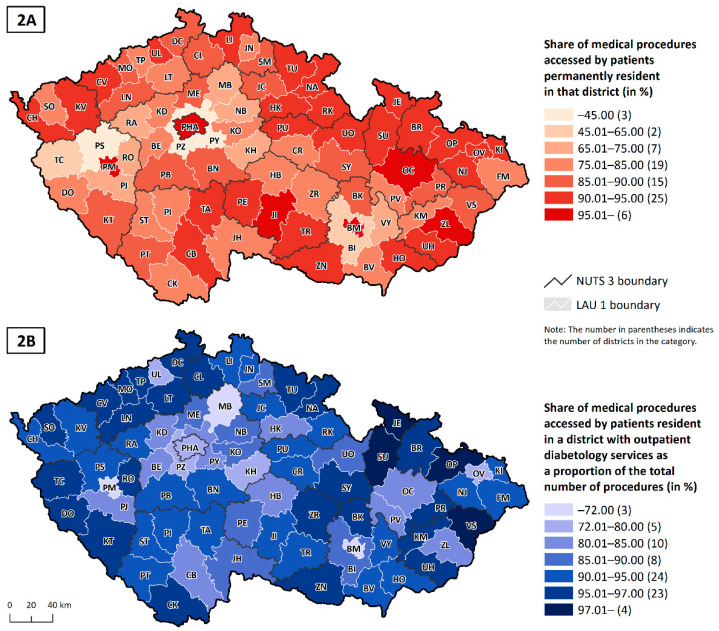
Share of medical procedures accessed in the patient’s district of permanent residence (**2A**) and share of medical procedures accessed by patients resident in a district with outpatient diabetology, as a proportion of the total number of procedures (**2B**); Czechia, 2019, GHIC patients.

The share of procedures patients access in their district of residence is a very useful indicator for analysing regional differences and identifying which districts patients travel to for diabetology care (Figure 2A). One such type of district is that found in areas around large regional centres such as the capital Prague (PHA)—the outlying districts of Praha-západ (PZ) and Praha-východ (PY); or in Plzeň, the suburb of Plzeň-sever (PS), adjoining the city centre Plzeň-město (PM); or in Brno, the district of Brno-venkov (BV) that forms a ring around the centre Brno-město (BM). For example, 66.5% of medical procedures accessed by patients resident in the outlying Prague district of Praha-západ (PZ) were accessed in the city centre, 50.3% of the medical procedures accessed by patients resident in the outlying district of Praha-východ (PY) were located in the city centre of Prague (PHA) and 58.7% of procedures accessed by patients resident in the outlying district of Plzeň-sever (PS) were accessed in the city centre, Plzeň-město (PM). The healthcare services available in these city centre districts reflect this demand: healthcare capacity is well above the average value, when calculated per head on a district basis.

The second type of district is a geographically peripheral district with a low level of service access. These districts have difficulty attracting medical services to the area but are close enough to a regional centre for patients to be able to travel there several times a year. This set includes districts such as Tachov (TC), Kutná Hora (KH), Rakovník (RA) and Kroměříž (KM). There is a third set of districts that is interesting: districts located in more distant areas, often in the border areas. In many of these districts, healthcare actors make an effort to attract healthcare providers into the district, even if it is just for a few hours per week. One reason for this is that these districts tend to have a larger proportion of older people, who have difficulty accessing more distant regional centres. That is why districts such as Náchod (NA), Jeseník (JE) or Bruntál (BR), which are characteristically highly peripheral districts, have a higher proportion of patients accessing healthcare in their district of residence than is the case in the previous set of districts.

As has already been noted, in many districts with an important regional town, service provision reflects demand, which is higher because of the greater interest from patients in the surrounding districts. Although these districts provide care to the vast majority of their residents (see Figure 2A), the results for procedures performed show that these patients account for less than 80% of the total number of procedures (e.g., the city centre districts of Praha (PHA) and Brno-město (BM); the city centre Plzeň-město (PM) is an extreme example, with just under 60% of procedures being accessed by the district population (see Figure 2B). By contrast, in districts that are not located near an important regional centre and which have adequate healthcare provision, most of the performed services belong to the inhabitants of that district.

In addition to the characteristics of the districts themselves associated with their location, we can try to explain regional differences using other variables. Out of consideration for the available data, we selected several indicators to which we applied a simple correlation analysis. The aim was to determine whether statistically significant relationships could be found for the selected variables with the indicator Share of medical procedures accessed in the patient’s district of permanent residence (see Table 2).

The first group represents indicators related to both the sociogeographic characteristics of the districts and the density of the network of outpatient diabetology facilities. Indicators that indicate a higher proportion of the urban population emerged as statistically significant associations. It was found that the higher the level of urbanization, the lower the share of procedures reported to patients living in rural areas, the higher the share of procedures drawn in these districts. The highest level of association in this group is indicated by the variable share of procedures reported to patients who live in the municipality with outpatient diabetes. This indicator represents whether the patient can potentially receive healthcare in the municipality of residence. According to the results, if the diabetology provider is in the municipality of the patient´s residence, patients generally use it. It is, of course, related to the size of the municipality and the distribution of not only health services. Overall, we can conclude that patients living in municipalities with a higher number of inhabitants generally have more accessible outpatient diabetology services, and thus also use these services quite often in their areas.

The second group of variables concerns the capacity provided for outpatient diabetology in a study district, with respect to the amount and age structure of physicians. The results show that the higher the population per FTE (which indicates de facto insufficient medical capacity and a low possibility of choosing the provider), the more often patients receive services in another district than the one in which they live. The age structure of doctors also plays a role. The share of capacity of physicians aged under 40 years does not play a significant role, which may be due to, for example, the fact that doctors are still establishing their clients, they are close to other physicians such as general practitioners who can have a compensated diabetic patient in their care. On the contrary, a stronger relationship between patients and physicians can be expected in districts with a higher proportion of older physicians, given the longer-term care of the patient, and therefore a lower need for the patient to seek care outside their region of residence (the correlation is significant only at the 0.05 level).

The third group of indicators focuses on the procedures reported with regard to the demographic structure of patients. All the selected indicators have a significant correlation. Commuting outside the district of residence is associated with a higher number of reported procedures and a higher proportion of procedures for male patients. Conversely, as the age of the patient rises, the share of medical procedures accessed in the patient’s district of permanent residence increases (as illustrated in Figure 1A,B). 

**Figure 3:**
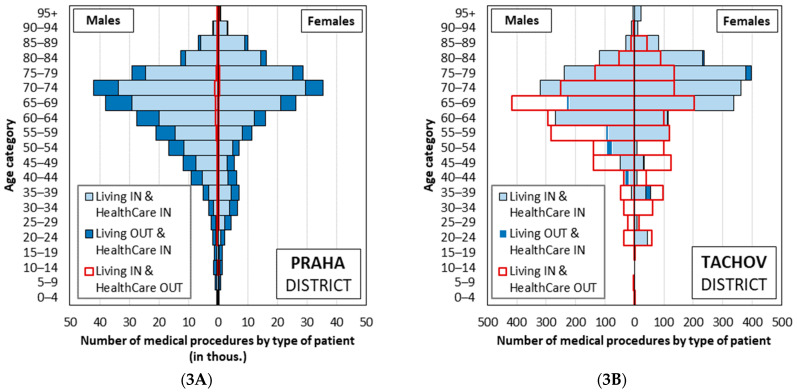
Comparison of the number of procedures performed for Praha district (**3A**) and Tachov district (**3B**) by patient type based on the place of residence and the location of outpatient diabetology provision; Czechia, 2019, GHIC patients. Note: **Praha district**: representative of a district characterised by a high number of incoming patients receiving care in that district and where the absolute minimum number of patients who live in that district travel outside the district for care. **Tachov district**: representative of a district characterised by a high number of patients travelling outside the district for care, and where at the same time a minimum number of patients residing in other districts come for care.

The patient population structure of these districts, or of healthcare provided, varies significantly. We can illustrate the two different types (see Figure 3) by comparing Prague city centre, Praha (PHA), with the district of Tachov (TC), located in the Plzeň region. Praha (PHA) overwhelmingly provides outpatient diabetology services to its own population (97% of procedures accessed by Praha (PHA) inhabitants are provided in Praha (PHA)). However, this figure accounts for just under 75% of the total number of procedures. That means that a quarter of the care is provided to inhabitants of other districts, mostly from the outlying Prague districts of Praha-západ (PZ) and Praha-východ (PY) (about 5% for each district). By contrast, the data for the district of Tachov shows that 50% of care accessed by its residents is located in a district other than Tachov (most frequently Plzeň-město, 35%). A very small proportion of patients travel into Tachov for care: just under 4% of all outpatient diabetology services are accessed by residents of other districts.

## 4. Discussion

In Czechia, as in other European countries, an adequate health care system should be available to all inhabitants. Simultaneously, the adequate network of health services should be effective. Policymakers need detailed information to establish the appropriate policy and evaluate it. The success of applied strategies depends on consideration of various factors in particular: patient needs, demographic structure of the inhabitants and the medical workforce, health system characteristics, finance strategy, and the whole situation in society [31,32]. Moreover, it is necessary to consider the circumstance of the physician´s choice to locate to certain regions. Physicians, especially young beginning physicians, usually prefer to work in urban areas rather than in the countryside. This and other reasons, lead to a higher proportion of older physicians in rural and socioeconomically challenged regions, which appears to create a potentially high risk to maintaining adequate health care in these areas. The increasing urban concentration of physicians, chiefly specialists to whom the diabetologist belongs, was observed not only in Czechia [31,32,33,34,35,36]. Our study confirms the uneven distribution of diabetology services that could be related to the factors discussed.

When assessing healthcare accessibility in a region, it is important to know the amount of healthcare provided linked to the patient’s place of residence, and/or the location of healthcare. The most frequently used indicators do not contain this information. For most indicators, the effectiveness of healthcare provision is calculated as number of inhabitants/patients in the given area (e.g., number of inhabitants per doctor, or per FTE). Therefore, if the data allow it, the researcher must dig deeper and attempt to analyse the values, not according to the patient’s place of residence but the location of healthcare.

**Figure 4:**
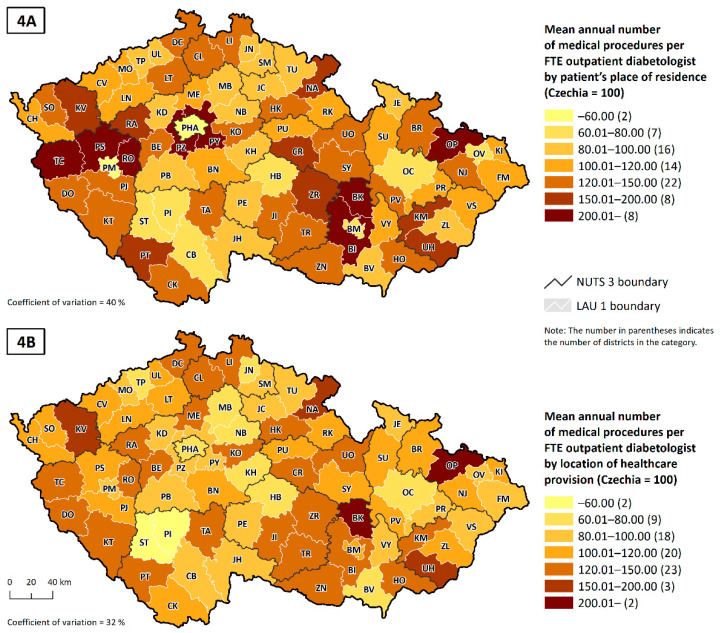
Mean annual number of medical procedures per FTE outpatient diabetologist by the patient’s place of permanent residence (**4A**), and by the location of healthcare provision (**4B**); Czechia, 2019, estimate for all insurance holders. Note: estimates for all insurance holders are based on the share of GHIC insurance holders as a proportion of the total number of insurance holders in that district by gender and five-year age range. To estimate the total number of procedures performed, we assumed that take-up by age and gender of insurance holder was the same regardless of health insurance provider.

To obtain a better understanding of this issue, we can compare two indicators of the average number of procedures per FTE outpatient diabetologist. The first indicator tells us the number of procedures per patient permanently resident in that district (Figure 4A), and the second tells us the number of procedures per patient receiving healthcare in that district (Figure 4B). A visual comparison of the two cartograms shows that using the second (more accurate) indicator reduces the regional differences in the phenomenon being observed, especially in core–peripheral areas, that is, districts with large city populations, such as the capital city, Praha (PHA), or the regional capitals of Plzeň-město (PM) and Brno-město (BM). 

Assessments of doctors’ workloads should take into account the true amount of healthcare accessed by all patients within that healthcare catchment area. That does not, however, mean that the patient’s place of residence is not important. Where there are areas that are known to have a lower rate of healthcare take-up, that may be due to a lack of healthcare capacity or the quality of the provision. It was the lack of capacity, expressed both in terms of the number of inhabitants per FTE and the average number of procedures per FTE, that emerged in the statistical analysis as one of the variables with the strongest correlation with the share of medical procedures accessed in the patient’s district of permanent residence (see Table 2). Unfortunately, it is not possible to identify indicators of the quality of services provided from the available data that could better explain some of the spatial linkages. For this purpose, it might be appropriate to conduct qualitative research on the patient’s reasons for commuting to health services. This would confirm the willingness of younger patients to travel longer distances for better quality services or insufficient capacity for services in the region of residence. This targeted analysis should be conducted so that health insurers, for example, who pay for the healthcare services via a public insurance system can identify such problems. Steps should be taken when drawing up policy agreements to ensure that attention is focused on weak points and on increasing medical capacity in districts where patients have to travel to access healthcare.

Capacity indicators are not the only factors that influence the assessment of health service utilization in the region of residence or beyond. With the example of outpatient diabetology services, it was confirmed that the demographic structure of patients in the region plays an important role. Patients living in regions where a higher proportion of reported procedures was reported for women, as well as for elderly patients, receive more healthcare in their region of residence. Men are more likely to travel more frequently and further than women, not only for health services [36,37,38]. Sex differences in mobility, especially work mobility, are put in the context of household roles and family situation [39,40]. Simultaneously, mobility of younger people is significantly higher in general [36]. It is usually related to commuting for study, work, and other services [41]. On average, personal mobility declines after reaching retirement age, mainly due to missing work mobility. Older people still travel for shopping, leisure trips, and visiting relatives [42], however, these activities are usually not so far from their place of residence. It is possible to assume that younger patients combine regular doctor visits with other activities outside of their municipality of residence in the place where they spend most of their time.

As previous studies show, the type of region (rural vs. urban) and the size of the municipality have a significant impact on healthcare utilization [43]. As mentioned above, health services, especially specialist care, are in large municipalities. Although most people are willing to travel for health services outside their place of residence, their willingness decreases with increasing distance and time spent on the road [6]. It is possible to deduce that patients who live in the municipality with the diabetology provider will rather use services in that area, which has been confirmed. In addition, patients from small municipality or rural areas have a significantly higher probability of travelling for healthcare in a different region. According to Goins et al. [44], rural inhabitants refer to the necessity of traveling outside of their municipality and insufficient transportation as one of the main barriers to suitable healthcare accessibility. In rural areas, there is usually limited public transport, so most people rely on car transport.

At the same time, it is important to mention another important factor; the position of the region in relation to the core areas. Core areas often affect the population of surrounding regions offering a high capacity for (not only) provided health services. These areas are mostly places of work, study, entertainment, and social life, where people spend most of their time. Due to this, people often access services, such as healthcare services, in the city centre, although they live outside the city [45,46]. Mostly, the greater the differences between the core and peripheral areas, the more significant commuting to the centres is [47]. Important cities with large populations, such as Prague, Brno and Plzeň, are some of the significant core areas which are frequently the target of commuting, as evidenced by our study.

Lastly, it is necessary to note that the temporary place of residence and permanent place of residence do not always coincide. In Czechia, the existing register only gives the inhabitant’s address of permanent residence; information is not kept on the temporary place of residence. The population census is an exception, but the data on individuals in the most recent census conducted in 2011 cannot be linked with, for example, the health registers. It is, however, known that the NUTS 3 level data on permanent addresses do not match temporary addresses for 1 to 3% of inhabitants, potentially rising, in some areas, to 20% of inhabitants [45,46]. Unfortunately, various strategic and policy documents, including those relating to healthcare provision, rely on the only official available data source—permanent residences—and so one cannot avoid using that information source, despite there being a significant risk it does not reflect actual reality.

In addition, we would like to reflect on how the spatial patterns of diabetes behaviour described above may have been influenced by the ongoing COVID-19 pandemic. Although the impact of this pandemic on the uptake, provision and availability of healthcare is significant on a global scale [48,49,50], we believe that in the segment of outpatient diabetology care in Czechia there are no significant changes in trends in the amount of healthcare provided, and patient behaviour from a longer term perspective. We base our claim on two facts; first, we rely on publicly available data, which is very limited for the COVID-19 period and is only in aggregate form. It appears that the impact of the pandemic on the uptake of outpatient diabetes services was small in 2020 (no data are yet available for 2021). Data from the annual reports of the largest health insurer company (which provided data for this study) show that the number of contacts, the number of points reported for health services, the total costs of medicines and medical devices, increased year-on-year (see Table 3) [51]. In our view, this clearly suggests there has not been a decline in the uptake of outpatient diabetes services.

The second fact concerns the network of outpatient diabetes service providers. This network is relatively stable in Czechia [29] and the uptake of services by these providers has been constant in the long term [16,28,36]. Moreover, it is clear from available studies that Czech patients are not very accustomed to changing their treating physician, and their willingness to travel longer distances for care is not very high and decreases with age (see [36]). In consideration of the older structure of diabetic patients (see Figure 1A), it can be suggested that the travel distance to the diabetology services has probably not increased during the pandemic. On the contrary, it seems likely that patients who had been commuting for care outside their region of residence have changed their treating physician to a physician from their region of residence, during the pandemic to reduce their movement and contacts with people. However, we encounter capacity limits. As can be seen in Figure 4, peripheral and rural areas have already had a higher intensity of care per FTE, so it is highly likely that doctors in these areas no longer have the additional capacity to treat new patients who have previously received care in another region. Other factors, such as the temporary closure of doctors’ offices due to political restrictions and illness of staff, could affect the use of healthcare services for individuals. Nevertheless, it should not have a significant impact on the long-term general results. We believe that our results are applicable to the Czech setting and patients using outpatient diabetes services, even during the COVID-19 pandemic. However, we are aware that in some other segments of health care or in other regions [50,52,53] the impact of this pandemic may have a non-negligible effect, not only on the spatial patterns of health service use.

## 5. Conclusions

Spatial healthcare accessibility is an important aspect of the provision and take-up of healthcare services, but is frequently overlooked in assessments of regional differences (largely because of the general lack of access to the necessary input data). Patients are not obliged to seek healthcare within their place of permanent residence and often travel to access healthcare outside their catchment area, in a different administrative area (district, region). This can lead to a situation in which two seemingly comparable regions, with similar population structures, in terms of number and structure of permanent residents and capacity of healthcare provision, turn out to serve diametrically different numbers of patients in reality. One area might be a ‘healthcare exporter’, where the amount of healthcare provided to inhabitants of other districts accounts for perhaps a quarter of all healthcare provision, while the other area might be a ‘healthcare importer’, where residents travel elsewhere for healthcare. Typical healthcare exporter areas are cities (local centres), whereas their surrounding areas (often the suburbs where residents commute into the centre and use its healthcare services) are often healthcare importers. There are various reasons why people access healthcare outside their place of permanent residence, not just practical ones (place of work or study), such as the desire to access quality healthcare or because of a lack of healthcare capacity.

Spatial healthcare accessibility is both geographically and demographically distinct. In analyses such as this one, it is important to bear in mind that two regions with marked differences in population composition may also differ in terms of healthcare demand. The ability of the population to travel for healthcare changes with age. For this article, however, and at the LAU 1 administrative level, differences in age structure that could affect level of healthcare take-up are not important. The key factor is the character of the area and its location vis-à-vis important local/regional centres. This should always be considered when assessing regional differences in healthcare accessibility.

## Figures and Tables

**Table 1:** Structure of patient population and medical procedures by the location of outpatient diabetology service; Czechia, 2019, GHIC insurance holders.

Administrative Unit	Number	Structure of Patient Population and Medical Procedures by the Location of Outpatient Diabetology Service (%)		
	Patients	IN	IN and OUT	OUT
municipalities (LAU 2)	207,240	87.8	1.3	10.9
districts (LAU 1)	366,537	87.0	1.1	11.9
regions (NUTS 3)	366,537	93.4	0.7	5.9
	Medical Procedures	IN	IN and OUT	OUT
municipalities (LAU 2)	1,408,073	86.7	2.2	11.1
districts (LAU 1)	2,481,129	85.8	1.9	12.3
regions (NUTS 3)	2,481,129	92.5	1.3	6.2

Key: IN = outpatient diabetology service in patient’s area of residence, IN and OUT = outpatient diabetology service in patient’s area of residence and in another location, OUT = outpatient diabetology service outside the patient’s area of residence. Note: At the municipal level (LAU2), only patients living in a municipality providing outpatient diabetology are taken into account.

**Table 2:** Dependence of selected variables on the indicator Share of medical procedures accessed in the patient’s district of permanent residence using Person’s correlation coefficient; Czechia, 2019.

Indicators	Pearson Correlation		Sig. (2-Tailed)
Indicators of district characteristics and density of outpatient diabetologists			
Population density (population per km^2^)	0.184		0.109
Urbanization rate (municipalities of 5000 inhabitants or more)	0.506	**	0.000
Share of procedures reported to patients living in municipalities of less than 2 thousand inhabitants	−0.432	**	0.000
Share of municipalities with outpatient diabetology	0.188		0.102
Share of procedures reported to patients who live in municipality with outpatient diabetology	0.573	**	0.000
Indicators of the medical capacity and its structure			
Number of inhabitants aged 15+ per FTE	−0.618	**	0.000
Share of the capacity of physicians aged under 40 years	0.105		0.362
Share of the capacity of physicians aged 60 and over	0.232	*	0.042
Indicators of reported medical procedures and their structure			
Average annual number of procedures per 1 patient	−0.526	**	0.000
Average age of the patient (weighted by number of reported procedures)	0.382	**	0.001
Share of procedures reported for patients aged 0–39 years	−0.244	**	0.032
Share of procedures reported for patients aged 40–64 years	−0.365	**	0.001
Share of procedures reported for patients aged 65 years and over	0.372	**	0.001
Masculinity index (number of procedures reported for male per 100 female procedures)	−0.362	**	0.001

Note: ** Correlation is significant at the 0.01 level (2-tailed); * Correlation is significant at the 0.05 level (2-tailed); N = 77 districts.

**Table 3:** Selected indicators of outpatient diabetes care in 2016–2020 for insured persons of the largest health insurance company (GHIC).

Indicators	2016	2017	2018	2019	2020
Number of Contacts between Patients and Providers	1,022,176	1,020,695	1,028,224	1,053,410	1,059,570
Number of Points (in thousands) *	395,422	406,653	414,291	438,812	449,906
Cost of Medicines and Medical Devices (in thousands CZK)	2,118,932	2,270,028	2,328,419	2,546,492	2,693,970

* Note: Each medical procedure is assigned a number of points, which reflects the complexity of the procedure, and the value of the point has its financial expression = the number of points multiplied by the value of the point subsequently determines the amount of reimbursement by the health insurance companies for the reported medical procedure.

## Data Availability

Not applicable.
